# Chlorophyll fluorescence as a light signal enhances iron uptake by the marine diatom *Phaeodactylum tricornutum* under high-cell density conditions

**DOI:** 10.1186/s12915-021-01177-z

**Published:** 2021-11-23

**Authors:** Xuehua Liu, Xiujun Xie, Shan Gao, Lepu Wang, Lu Zhou, Yao Liu, Qiang Hu, Wenhui Gu, Guangce Wang

**Affiliations:** 1 CAS and Shandong Province Key Laboratory of Experimental Marine Biology, Center for Ocean Mega-Science, Institute of Oceanology, Chinese Academy of Sciences, Qingdao, China; 2 Laboratory for Marine Biology and Biotechnology, Qingdao National Laboratory for Marine Science and Technology, Qingdao, China; 3 College of Earth Sciences, University of Chinese Academy of Sciences, Beijing, China; 4 Public Technology Service Center, Institute of Oceanology, Chinese Academy of Sciences, Qingdao, 266071 China; 5 Institute for Advanced Study, Shenzhen University, Shenzhen, China

**Keywords:** Chlorophyll fluorescence, Density-dependent, Iron uptake, Biotic signal

## Abstract

**Background:**

Diatoms usually dominate phytoplankton blooms in open oceans, exhibiting extremely high population densities. Although the iron uptake rate of diatoms largely determines the magnitude and longevity of diatom blooms, the underlying mechanisms regulating iron uptake remain unclear.

**Results:**

The transcription of two iron uptake proteins, ISIP2a and ISIP1, in the marine diatom *Phaeodactylum tricornutum* was enhanced with increasing cell density, whereas the cellular iron content showed the opposite trend. When compared with the wild-type strain, knockdown of *ISIP2a* resulted in 43% decrease in cellular iron content, implying the involvement of ISIP2a in iron uptake under high-cell density conditions. Incubation of the diatom cells with sonicated cell lysate conditioned by different cell densities did not affect *ISIP2a* and *ISIP1* expression, ruling out regulation via chemical cues. In contrast, *ISIP2a* and *ISIP1* transcription were strongly induced by red light. Besides, chlorophyll fluorescence excited from the blue light was also positively correlated with population density. Subsequently, a “sandwich” illumination incubator was designed to filter out stray light and ensure that the inner layer cells only receive the emitted chlorophyll fluorescence from outer layers, and the results showed that the increase in outer cell density significantly elevated *ISIP2a* and *ISIP1* transcription in inner layer cells. In situ evidence from Tara oceans also showed positively correlated between diatom ISIP transcripts and chlorophyll content.

**Conclusions:**

This study shows that chlorophyll fluorescence derived from neighboring cells is able to upregulate *ISIP2a* and *ISIP1* expression to facilitate iron assimilation under high-cell density. These results provide novel insights into biotic signal sensing in phytoplankton, which can help to elucidate the underlying mechanisms of marine diatom blooms.

**Supplementary Information:**

The online version contains supplementary material available at 10.1186/s12915-021-01177-z.

## Background

Iron, an essential trace element with multiple biological functions [1], presents low bioavailability across high-nutrient low-chlorophyll (HNLC) regions, which restrains the growth of most of the phytoplankton [2, 3]. Diatoms often dominate the phytoplankton communities in HNLC regions owing to their ability to survive in chronically iron-limited waters and take advantage of pulsed iron supplies [2–5]. During natural phytoplankton blooms when extracellular iron is sufficient, the phytoplankton population density mostly reaches 10^6^ cells/L [6–8], sometimes even as high as 3×10^8^ cells/L in the HNLC regions, such as the South Pacific Ocean, North Pacific Ocean, and Indian Ocean et al. [9]. Such explosive increase in a cell population can result in increased inter- and intraspecies competition for iron. Under such circumstances, the rate of iron uptake predominantly determines the composition, dynamics, magnitude, and longevity of the algal bloom [3, 10].

Marine diatoms have been demonstrated to possess an efficient iron uptake system mediated by a wide range of proteins, especially ISIP2a and ISIP1 [1, 11–13]. Both ISIP2a and ISIP1 are widely distributed in the genomes, metagenomes, and meta-transcriptomes of numerous marine algal species and communities [12–15]. ISIP2a, a phytotransferrin, is responsible for high-affinity inorganic dissolved iron acquisition, and its absorption of dissolved ferric iron is coordinated with carbonate anion [13, 16]. Besides, ISIP1 mediates a siderophore uptake system with a high affinity for iron acquisition in diatoms [12]. As the two most predominant proteins in the iron uptake system, ISIP2a and ISIP1 play important roles in the primary response to iron deficiency, resulting from multiple environmental factors.

Previous studies have comprehensively discussed the physical and chemical factors controlling the iron uptake process by phytoplankton, including the total iron concentration in the environment, high light, low temperature, and pH [17–20]. Therefore, marine diatoms could directly respond to environmental iron changes. On the other hand, could these “smart” cells percept the biological factors such as population cell density and regulate the iron uptake indirectly? Furthermore, what are the underlying regulatory signals?

As the primary source of energy for photosynthetic organisms, light is also a key carrier of information from the surrounding environment [21, 22]. Light-sensing and light regulation driven by complicated light-regulatory mechanisms are believed to strongly contribute to phytoplankton perception of the marine environment [21, 22]. During algal blooms, the increase in phytoplankton population density results in the spatial and temporal variability of the light spectra underwater, which may have an influence on the physiological process of phytoplankton. Given the contribution of chlorophyll fluorescence to red and far-red irradiation underwater, especially during blooms, recent studies have proposed that chlorophyll fluorescence may be an information carrier underwater [23, 24]. Phytoplankton has been proposed to have the ability to sense chlorophyll fluorescence from neighboring phytoplankton cells, thus partitioning the space at microscale [23, 24]. Besides, as a potential signal that can be influenced by cell density, is chlorophyll fluorescence related to the iron uptake process during diatom blooms in HNLC regions?

The present study aimed to elucidate the relationship between iron uptake pattern and cell density in *Phaeodactylum tricornutum*, a representative marine diatom with a solid background for iron uptake research. To investigate the signaling factor in the iron uptake regulation, the potential role of light, especially chlorophyll fluorescence, was investigated. Our results lay the foundation for the newly biotic signal sensing in phytoplankton, which would be helpful to elucidate the multiple ecological events, especially diatom blooms.

## Results

### Physiological response of Phaeodactylum tricornutum to high-cell density

**Fig. 1:**
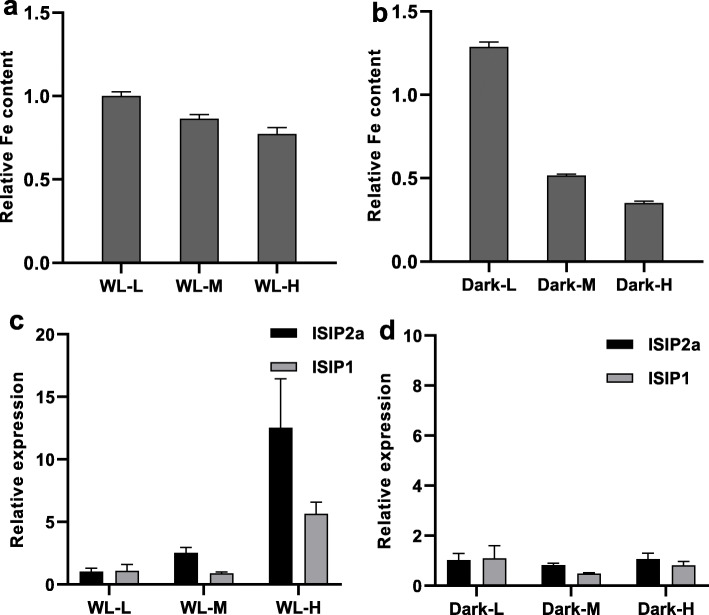
The effect of different cell densities on the relative intracellular Fe content and relative expression of *ISIP1* and *ISIP2a* of *P. tricornutum*. The cells were adjusted to different cell densities (L for low-cell density, M for middle cell density, and H for high-cell density) and cultured at white light (WL) and dark (Dark) for 24 h. Then, intracellular Fe content with different cell densities was determined under white light (WL, **a**) or dark conditions (Dark, **b**) and normalized by WL-L Fe content. The relative expression of *ISIP1* and *ISIP2a* of *P. tricornutum* with different cell densities were plotted under white light (WL, **c**) or dark conditions (Dark, **d**). Data was shown as mean values ± SD for three independent experiments

The effects of different cell densities on the intracellular iron concentration in *P. tricornutum* were examined under WL and dark conditions using ICP-OES. Under WL conditions, the relative intracellular iron content decreased with the increasing cell density (Fig. 1a). A similar trend was also observed with respect to the relative intracellular iron content under dark conditions (Fig. 1b). However, at the same high-cell density, the relative intracellular iron concentration under dark conditions was significantly lower than that under WL conditions (Fig. 1a, b).

To examine the response of the iron uptake system to cell density, the expression levels of *ISIP2a* and *ISIP1*, encoding the two most abundant proteins produced in iron starvation states, were analyzed [11, 16]. As shown in Fig. 1c, high-cell density induced the expression of *ISIP2a* and *ISIP1* under WL conditions, demonstrating their important role in iron uptake under high-cell density conditions.

Photosynthetic organisms require high-iron content to effectively sustain their photosynthesis, because about half of their intracellular iron is bound to photosystem proteins [25]. In the present study, a chlorophyll fluorescence assay was performed to evaluate the photosynthetic efficiency of *P. tricornutum* under light and dark conditions. The reduction in F_v_/F_m_, Y(II), rETR(II), and increased NPQ were a common response to the increased cell density under WL conditions (Table 1), reflecting reduced rETRs, increased NPQ, and compromised PSII reaction centers under high-cell density conditions. Besides, the Chl a content and DEPS decreased with the increasing cell density under WL conditions (Table 1). In contrast, under dark conditions, the photosynthetic parameters and DEPS did not exhibit any significant response to changes in the cell density. At similar high-cell density, the values of photosynthetic parameters and Chl a content under WL conditions were higher than those under dark conditions.

**Table 1:** Photosynthetic performance, pigment content of *P. tricornutum* and Fe concentration of medium under different cell density

		Dark-L	Dark-M	Dark-H	WL-L	WL-M	WL-H
**Fv/Fm**		0.35±0.04	0.38±0.01	0.33±0.02	0.6±0.03	0.59±0.02	0.58±0.01
**Y(II)**	PAR: 86 μE/m^2^ s	0.24±0.02	0.23±0.01	0.24±0.01	0.59±0.01	0.57±0.01	0.54±0.01
	PAR: 611μE/m^2^ s	0.16±0.04	0.16±0.02	0.16±0.02	0.4±0.03	0.38±0.01	0.35±0.02
**rETR(II)**	PAR: 86 μE/m^2^ s	8.77±2.06	9.37±0.57	7.65±1.34	20.3±0.52	20.5±0.17	19.67±0.29
	PAR: 611 μE/m^2^ s	42±11.19	40.47±5.46	41.2±3.99	101.67±7.77	96.6±1.61	90.43±4.35
**NPQ**	PAR: 86 μE/m^2^ s	1.05±0.1	1.09±0.13	1.29±0.2	0.04±0.07	0.03±0.02	0.05±0.03
	PAR: 611 μE/m^2^ s	0.9±0.09	0.93±0.02	1.08±0.13	0.09±0.09	0.1±0.03	0.19±0.04
**PSII rETRmax (μmol/m**^**2**^**s)**		84.35±5.3	55.15±23.26	58.2±5.33	114.1±6.84	106.43±4.8	101.53±5.12
**PSI rETRmax (μmol/m**^**2**^**s)**		102.8±59.54	432.55±405.95	444.07±43.13	183.33±47.59	200.63±31.22	156.65±46.32
**chla content mg/g DW**		0.85±0.08	1.17±0.01	0.91±0.02	1.18±0.01	0.88±0.01	0.51±0.04
**DEPS**		0.29	0.32	0.20	0.15	0.14	0.09
**Fe content (μM)**	ASW +f/2: 11.62±0.04	8.31±0.08	8.34±0.14	8.20±0.05	8.77±0.1	8.78±0.27	8.80±0.11

Data was shown as mean values ± SD for three independent experiments

### ISIP2a and ISIP1 facilitate iron absorption under high-cell density conditions

**Fig. 2:**
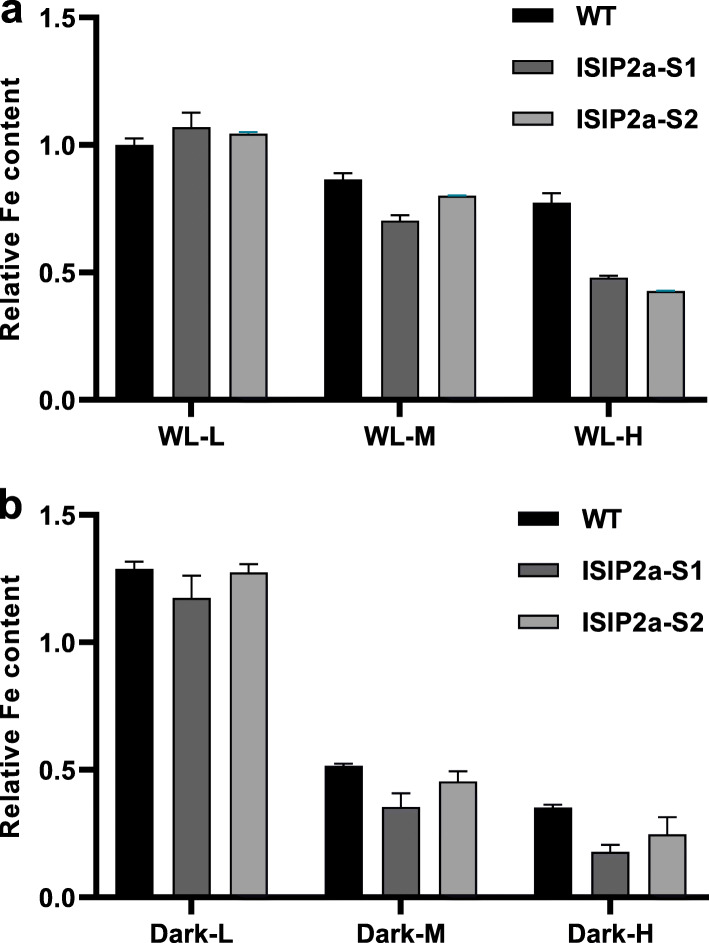
Relative intracellular Fe content of ISIP2a-S1 and ISIP2a-S2 under different cell densities. L for low-cell density, M for middle cell density, and H for high-cell density. The cell was incubated for 24 h under white light (**a**) or dark conditions (**b**). Intracellular Fe content was normalized by WL-L group of WT. Data was shown as mean values ± SD for three independent experiments

To further investigate the potential role of *ISIP2a* under high-cell density conditions, the *ISIP2a* knockdown strains were constructed. RNAi suppression of *ISIP2a* transcript levels in the ISIP2a-S1 and ISIP2a-S2 mutants were confirmed by qRT-PCR (Additional file 1: Fig. S1b). The growth characteristics of ISIP2a-S1 and ISIP2a-S2 mutants were determined under iron-replete and iron-deplete conditions. As shown in Additional file 1: Fig. S1a, there were no significant growth differences among wild-type, ISIP2a-S1, and ISIP2a-S2 strains under iron-replete conditions. However, ISIP2a-S1 and ISIP2a-S2 strains showed a significantly slower growth rate than the wild-type strain, which further confirmed the RNAi suppression effect in ISIP2a-S1 and ISIP2a-S2 mutants. As presented in Fig. 2a, the relative cellular iron content in ISIP2a-S1 and ISIP2a-S2 mutants was much lower than that in WT strain under the same cell density. The relative cellular iron content in ISIP2a-S1 under high-cell density showed a 42.74% decrease, compared to WT. A similar trend was observed under dark conditions (Fig. 2b). These results demonstrated that *ISIP2a* is crucial for iron absorption under high-cell density, both in WL, and dark conditions.

### Effect of light on the regulation of ISIP2a and ISIP1 expression

**Fig. 3:**
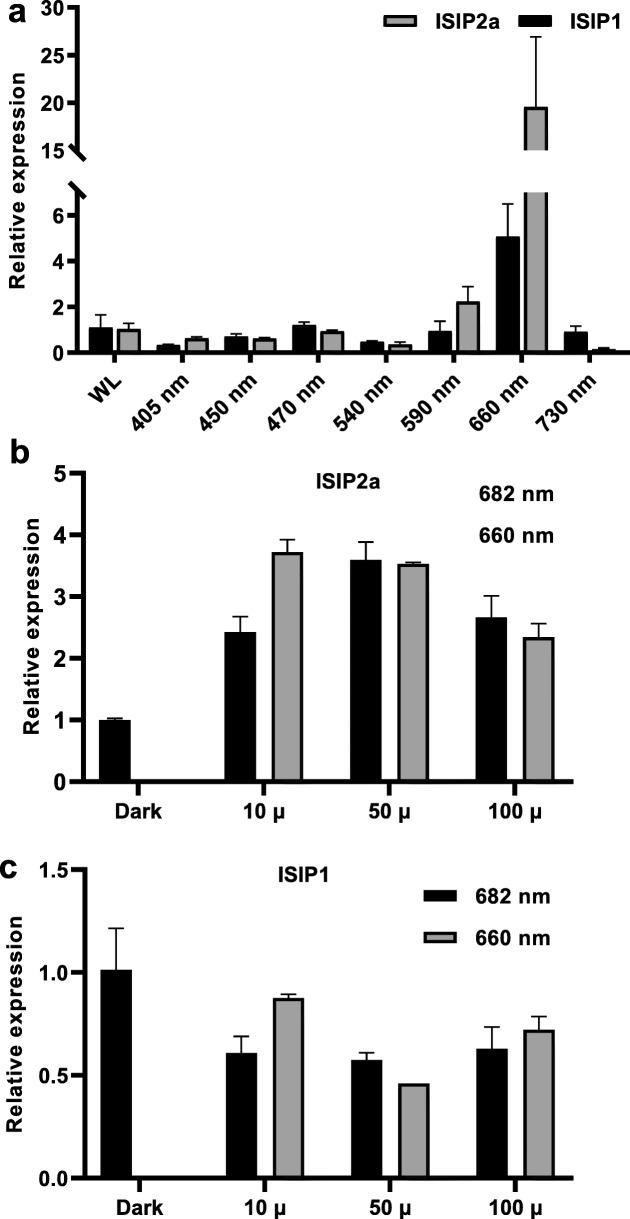
The effect of light wavelength on the *ISIP2a* and *ISIP1* expression. **a** After a 24-h illumination at 80 μmol/m^2^/s under different light wavelength in multi-cultivator MC 1000, the relative expression of *ISIP2a* and *ISIP1* were determined using qRT-PCR. **b**, **c** After illumination under different light intensity (100 μmol/m^2^/s, 50 μmol/m^2^/s, 10 μmol/m^2^/s) for 24 h, the relative expression of *ISIP1* (**b**) and *ISIP2a* (**c**) was determined by qRT-PCR. Data was shown as mean values ± SD for three independent experiments

To ascertain the effect of light on inducing *ISIP2a* and *ISIP1* expression, the expression levels of *ISIP2a* and *ISIP1* were measured at eight different wavelengths. The results showed that *ISIP2a* and *ISIP1* were significantly upregulated at 660 nm (red light), whereas the expression levels of these genes at other wavelengths were similar to those noted under WL conditions (Fig. 3a). These findings suggested that red light can significantly induce the expression of *ISIP2a* and *ISIP1*. As chlorophyll fluorescence and Raman scattering are the source of red light in deep layers [24] and the increased cell density can inevitably change the chlorophyll fluorescence, we examined whether chlorophyll fluorescence can act as a light signal.

First, red light with a wavelength of 682 nm was used to simulate chlorophyll fluorescence. As shown in Fig. 3b, after 24 h of irradiation at 682 nm, the expression of *ISIP2a* was significantly increased along with different light intensities (10 μmol/m^2^/s, 50 μmol/m^2^/s), while the similar expression pattern was not exhibited by *ISIP1* (Fig. 3c).

**Fig. 4:**
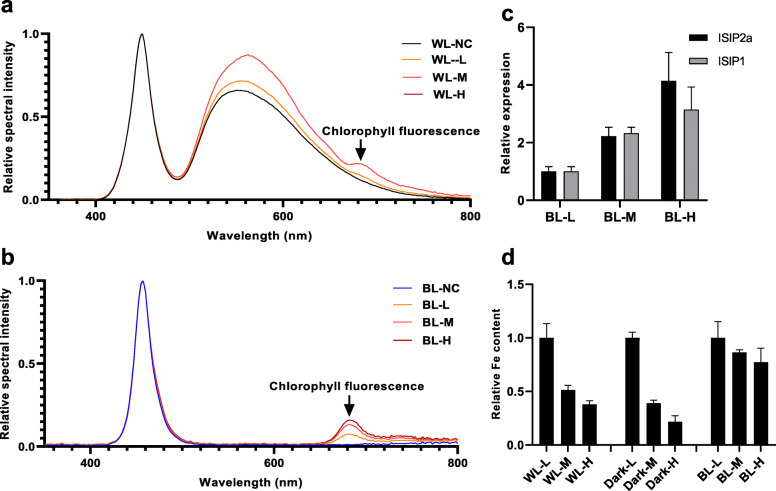
Chlorophyll fluorescence induced by white light (WL) and blue light (BL) at different cell densities. The excited light spectral of 1 mL cells under different cell densities (L, M, and H) were measured, with incident light of WL (**a**) or BL (**b**) at 80 μmol/m^2^/s. The excited chlorophyll fluorescence with a peak at 685 nm was pointed by a black arrow and the BL-excited chlorophyll fluorescence intensity was calculated and showed in Fig. 6. **c** The relative expression of *ISIP1* and *ISIP2a* under BL at different cell density. Cells were adjusted to different cell densities and cultured at BL for 24 h. **d** The relative Fe content under different cell densities was determined and normalized by the WL-L group. Data was shown as mean values ± SD for three independent experiments

Subsequently, endogenous chlorophyll fluorescence of *P. tricornutum* was induced using WL and BL. As indicated in Fig. 4a, b, both WL and BL could induce chlorophyll fluorescence, and the chlorophyll fluorescence intensity strengthened with increasing cell density. As BL failed to induce the expression of *ISIP2a* and *ISIP1* (Fig. 3a), but could stimulate the algal cells to emit different chlorophyll fluorescence intensities under various cell densities (Fig. 4b), we used BL to examine whether chlorophyll fluorescence is involved in the process of induction of *ISIP2a* and *ISIP1* in *P. tricornutum* under high cell density conditions. The results showed that *ISIP2a* and *ISIP1* expression increased with the increased cell density under BL, demonstrating that chlorophyll fluorescence induced the expression of *ISIP2a* and *ISIP1* (Fig. 4c). Also, under BL and WL conditions, the cellular iron content was relatively higher than that of dark conditions under high-cell density (Fig. 4d).

**Fig. 5:**
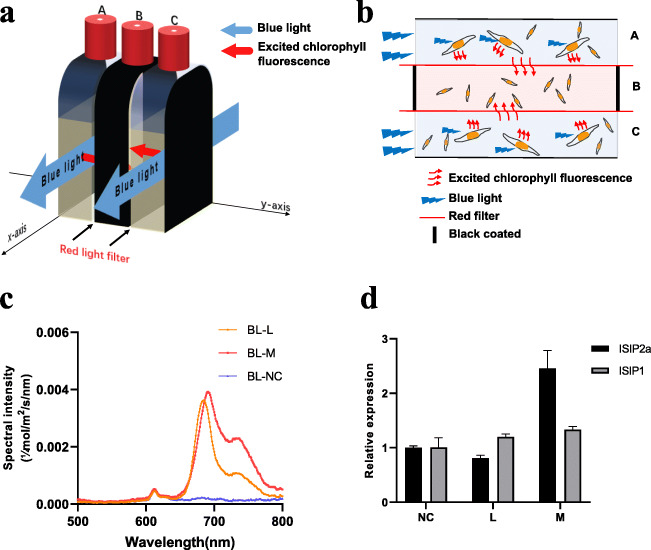
Relative expression of *ISIP2a* and *ISIP1* in the experiments carried out with the “sandwich” device. **a** “Sandwich” device configuration. By applying direct blue light at 80 μmol/m^2^/s on bottles A and C along with *x*-axis, chlorophyll fluorescence is excited in the *y*-axis. Due to the presence of the black coating and the red filter, the algae in bottle B can only receive light from the red zone in the *y*-direction. Changing the cell density in the A and C bottles (NC represents ASW, L represents low-cell density, and M represents middle cell density) can change the intensity of the excited fluorescence. **b** Side view of “sandwich” device. **c** The excited light spectral arriving in bottle B. **d** Relative expression of *ISIP2a* and *ISIP1*. Data was shown as mean values ± SD for three independent experiments

To further eliminate the involvement of chemical factors, we designed a “sandwich” device (Fig. 5) to separate the algal cells presenting endogenous chlorophyll fluorescence from irradiated cells. The *P. tricornutum* cells in bottle A and bottle C were excited by applying direct light in the *x*-axis to emit chlorophyll fluorescence in the *y*-axis (Fig. 5a, b). Owing to the presence of a black coating and red filter, the algal cells in bottle B could only receive light from the red zone in the *y*-axis. By altering the cell density in bottles A and C, the intensity of the excited fluorescence could be changed. As shown in Fig. 5c, d, the expression levels of *ISIP2a* and *ISIP1* in the irradiated cells slightly increased with the increasing density of cells, which was excited to emit chlorophyll fluorescence.

### Calculated chlorophyll fluorescence intensity in open oceans

**Fig. 6:**
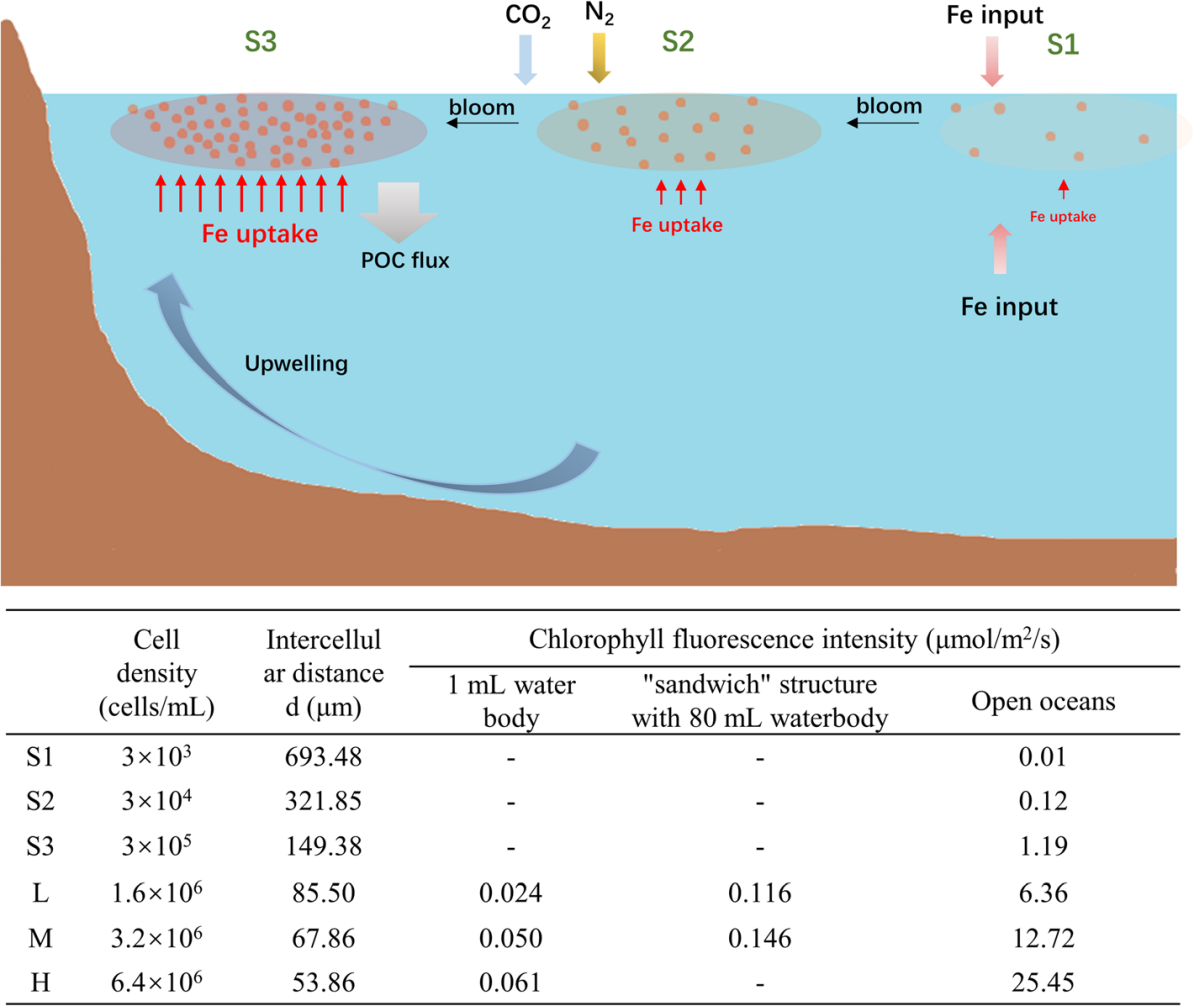
Schematic illustration of the bloom process in natural oceans and calculated chlorophyll fluorescence intensity. S1, S2, and S3 represent three stages with different cell density, during blooms accompanied with huge biomass and large-scale in actual oceans. The intracellular distance was calculated by cell density. Chlorophyll fluorescence intensity in 1 mL water body were measured according to light spectral in Fig. 4. Chlorophyll fluorescence intensity in “sandwich” structure with 80 mL waterbody according to light spectral in Fig. 5. Chlorophyll fluorescence intensity in open oceans with a large scale was calculated using the mathematical model

In order to calculate the chlorophyll fluorescence intensity received at the single site of each cell, we build a mathematical model based on the radius of plastid *r* and attenuation coefficient of light underwater *K*_*d*_. In cases of system containing two cells, with incident blue light at 80 μmol/m^2^/s (Additional file 1: Fig. S2), the chlorophyll fluorescence intensity varies from 0.01 to 3.61×10^-5^ μmol/m^2^/s with a cellular distance ranging from 0 to 100 μm (Additional file 1: Fig. S2), which is too weak compared with that in “sandwich” structure (Fig. 6), suggesting the limited possibility of chlorophyll fluorescence perceived by a cell.

Furthermore, considering the huge biomass and large-scale during diatom blooms, we build another mathematical model to calculate chlorophyll fluorescence intensity under stages with different cell densities (S1, S2, and S3) during blooms in actual oceans (Fig. 6, Additional file 1: Fig. S3). The calculated chlorophyll fluorescence intensity varies from 0.01 to 1.19 μmol/m^2^/s in S1, S2, and S3 stages. Also, in the cases of L, M, and H, the chlorophyll fluorescence intensity strengthened with elevated scale, that is, the total diatom biomass (Fig. 6). As the distance R increases, the contribution of cells to CFPF is rapidly reduced. Cells within 12.10 m contribute 99.9% of the total CFPF in a population, regardless of cell density (Additional file 1: Fig. S3).

### The ISIPs expression in metatranscriptome (MetaT) with respect to chlorophyll content

**Fig. 7:**
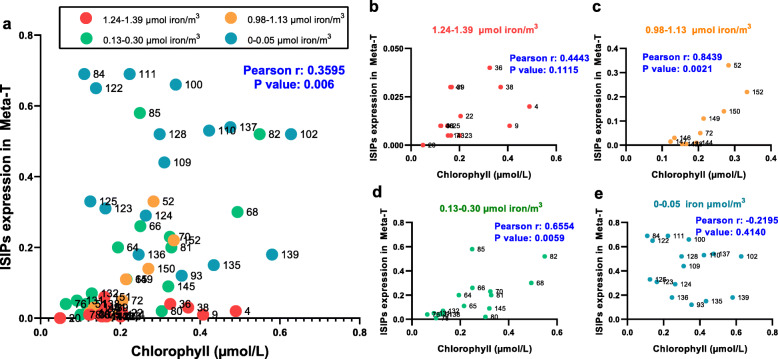
The *ISIP* expression in metatranscriptome (MetaT) with respect to chlorophyll content. *ISIPs* in each Tara station were expressed as a percentage of the total value of *ISIP1*, *ISIP2a*, *ISIP2b*, and *ISIP3* and normalized by the total diatom unigene expression, respectively. **a** Correlations between *ISIPs* expression in MetaT and chlorophyll content among various Tara stations. The station numbers are shown next to the circle and are colored according to the in situ measured iron concentration: red for 1.24–1.39 μmol iron/m^3^; orange for 0.98–1.13 μmol iron/m^3^; green for 0.13–0.30 μmol iron/m^3^; and blue for 0–0.05 μmol iron/m^3^. For Tara stations with in situ measured iron concentrations, the correlations between chlorophyll content and *ISIP* expression level were plotted in **b**, and **c**, **d**, and **e** follow suit. The Pearson correlation coefficients (Pearson r) and their statistical significance (*p* value) are indicated in each graph

The *ISIP* expression in Meta T showed weak positive correlations with chlorophyll content among various Tara stations (Fig. 7a). For Tara stations with in situ measured iron concentrations between 1.24 and 1.39 μmol iron/m^3^, the Pearson r between chlorophyll content and *ISIP* expression level was 0.44 with *p* value at 0.11, not significant enough (Fig. 7b). However, for Tara stations with in situ measured iron concentration at 0.98-1.13 μmol iron/m^3^ and 0.13–0.30 μmol iron/m^3^, the Pearson r between chlorophyll content and *ISIP* expression level reached 0.84 and 0.66, with *p* value at 0.002 and 0.001, respectively (Fig. 7c, d), an indication of the strong correlations between *ISIP* expression in Meta T and chlorophyll content in open oceans.

## Discussion

### Expression of iron uptake system in *P. tricornutum* is correlated with cell density

The results of the present study showed that *P. tricornutum* can sense high-cell density and induce *ISIP2a* and *ISIP1* expression to accelerate iron absorption, which is unrestricted by environmental iron concentrations. Considering that marine diatoms could be limited by low iron levels in seawater and that iron uptake proteins will be upregulated to accomplish rapid iron acquisition [1, 11–13], we first eliminated the effect of environmental iron concentration on the experiment by adding sufficient iron to the culture medium (11.7 μM, which is much higher than the iron content in the oceanic environment). After 24 h of treatment, the residual iron in the medium did not decrease with the increased cell density, which indicated that environmental iron was sufficient and ruled out the possibility that the high-population cell density might act on individual cells by removing iron from the medium (Table 1). Under such circumstances, along with increased cell density, *P. tricornutum* presented significantly elevated expression of iron uptake proteins, including ISIP2a and ISIP1 (Fig. 1), which suggested that the algal cells could perceive cell density and induce *ISIP2a* and *ISIP1* expression to accelerate iron absorption under high-cell density conditions. Furthermore, knockdown of *ISIP2a* in *P. tricornutum* significantly decreased the iron content under high-cell density conditions (Fig. 2), which further confirmed the role of *ISIP2a* in iron uptake at high-cell density. Although previous studies have proved that *ISIP2a* and *ISIP1* are responsible for iron absorption in marine diatoms and could be induced by low environmental iron concentrations [12, 13, 16], the other regulatory mechanisms of *ISIP2a* and *ISIP1* expression still remain unclear. The results of the present study suggested that cell density, in addition to the iron ions in the environment, also affects *ISIP2a* and *ISIP1* expression.

However, the intracellular iron content in *P. tricornutum* was significantly decreased under high-cell density conditions (Fig. 1), whereas the photosynthesis performance and Chl a content decreased with the increasing cell density (Table 1), thus suggesting that *P. tricornutum* cells may be iron-deficient under high-cell density conditions. As low cellular iron concentration is considered to induce the upregulation of iron uptake proteins for rapid iron uptake [1, 11–13], we investigated whether the upregulation of *ISIP2a* and *ISIP1* is induced by the low cellular iron concentration or directly regulated by cell density. In this study, the expression of flavodoxin in *P. tricornutum* showed no changes with the increasing cell density (Additional file 1: Fig. S4). Flavodoxin, a non-iron-containing protein that can replace ferredoxin to mediate electron transfer in a range of metabolic reactions under iron deficiency, is sensitive to iron starvation and is considered as an iron deficiency marker in phytoplankton [26, 27]. It has been reported that *P. tricornutum* could increase the expression of flavodoxin by 25–50-fold under iron-limited conditions [1]. Thus, a low level of flavodoxin indicated that the cells were not stressed by iron starvation under high-cell density conditions. Furthermore, the expression pattern of *ISIP2a* and *ISIP1* under WL and dark conditions was analyzed. Although the increase in cell density under both WL and dark conditions lowered the cellular iron concentration, *ISIP2a* and *ISIP1* were only upregulated under WL conditions (Fig. 1), thus indicating that intracellular low iron status is not exclusively regulated by *ISIP2a* and *ISIP1* expression and that light is necessary for *ISIP2a* and *ISIP1* regulation.

### Regulation of ISIP2a and ISIP1 expression in *P. tricornutum* under high-cell density is mediated by chlorophyll fluorescence intensity

We first evaluated the possibility of chemical cues involved in the *ISIP2a* and *ISIP1* expression regulation under high-cell density. The results in this study showed no obvious changes in *ISIP2a* and *ISIP1* expression under diverse sonicated cell lysate conditioned by different cell densities (Additional file 1: Fig. S5), which preliminarily ruled out the possible involvement of chemical cues in *ISIP2a* and *ISIP1* regulation. However, in bacteria, the production of siderophore, a component used for iron assimilation, has been reported to be density-dependent and regulated by quorum sensing [28, 29]. The bacterial cells can sense their population size to regulate siderophore synthesis [28, 29], which is mediated by chemical components produced and secreted by these cells [30–33]. Similar chemical cues have also been reported in marine diatom *Chaetoceros socialis*. Pelusi et al. used the *C. socialis* cell lysate conditioned by high-cell density and induced transformation from vegetative cells to resting spores, which proved the existence of density-mediated chemical signals [34]. In contrast, the results of the present study showed that the regulation of *ISIP2a* and *ISIP1* expression in the marine diatom *P. tricornutum* does not involve chemical signals and might be affected by other forms of signal factors.

Besides chemical cues, light has been proposed to be a signal used by diatoms to monitor and gain information about the surrounding environment (reviewed in [22, 35]). Recent studies have provided some evidence for the conjecture that chlorophyll fluorescence could be sensed as a light signal in phytoplankton [23, 24]. However, this speculation is preliminary and indirect owing to the lack of explicit confirmation. The results of the present study provide stronger evidence for the participation of light in the regulation of *ISIP* expression under different cell density conditions. First, although the iron content in cells decreased with increasing cell density both under light and dark conditions, the expression of *ISIP2a* and *ISIP1* was detected only in the presence of light (Fig. 1), demonstrating that light is involved in the response of *ISIP2a* and *ISIP1* to cell density. Second, red light (both 660 nm and 682 nm) could significantly induce the expression of *ISIP2a* in *P. tricornutum*, whereas the other lights, including WL and BL, showed no such effect (Fig. 3), which suggested that red light in seawater, such as chlorophyll fluorescence, may participate in the signaling pathway of *ISIP2a* and *ISIP1* induction under high-cell density conditions. Third, alteration in the cell density and illumination to change the excitation intensity of endogenous chlorophyll fluorescence revealed that *ISIP* expression was positively correlated with the chlorophyll fluorescence intensity (Figs. 1 and 4). To further eliminate the involvement of chemical factors, the cells exhibiting endogenous chlorophyll fluorescence were partitioned from the irradiated algal cells using a red filter (Fig. 5). With the increase in cell density on both sides, the chlorophyll fluorescence intensity of cells in the middle layer increased, along with slight upregulation of *ISIP2a* and *ISIP1* expression (Fig. 5). Overall, these results demonstrated that chlorophyll fluorescence probably is the signal for iron uptake in response to high-cell density.

Furthermore, multiple cis-acting elements involved in light-responsive were detected in the *ISIP2a* upstream region, especially the cis-acting element involved in DPH downregulation (Additional file 1: Fig. S6), suggesting the possibility that DPH could regulate *ISIP2a* expression. DPH is a widespread family of red/far-red responsive photoreceptors and was first characterized by Fortunato et al. in the marine diatoms *P. tricornutum* and *Thalassiosira pseudonana*, primarily demonstrating the red/far-red light sensing ability and far-red light signaling in marine diatoms [24]. Fortunato et al. knocked out the DPH in *P. tricornutum* and found that the red light-induced proteins were still upregulated under red light illumination, indicating that DPH does not play a role in red light signaling in marine diatom [24]. However, the phytochrome gene family contains three main clades encoding phytochrome A, B, and C, among which phytochrome A mediated the far-red light responses while phytochromes B and C mediated the red-light responses [36]. Thus, we speculated that there may be other unexamined DPH regulating the red-light responsive proteins in marine diatoms.

### Chlorophyll fluorescence in open oceans and its ecological implications

It has been indicated that red and far-red light from sunlight rapidly attenuate with increasing depth of the sea and can only be detected on the sea surface. However, a recent study proved that red and far-red waveband photons could be detected underwater, which was supported by chlorophyll fluorescence and Raman scattering [24]. Mathematical model results revealed that the chlorophyll fluorescence intensity perceived by cells is inversely proportional to the cellular distance, and directly proportional to the total population biomass, the latter is determined by cell density and bloom scale (Fig. 6). However, the contribution of cells beyond a radius of 12.1 m to the total calculated chlorophyll fluorescence intensity is negligible (Additional file 1: Fig. S3b). In large-scale diatom blooms in open oceans, calculated chlorophyll fluorescence intensity varies from 0.01 to 1.19 μmol/m^2^/s, roughly equivalent to the red-light intensity 10–20 m underwater, reported by Fortunato et al. [24], implying the chlorophyll fluorescence is perceptible in natural oceans.

In situ evidence derived from Tara oceans showed there is a positive correlation between MetaT expression of diatom *ISIPs* and chlorophyll content, especially when the iron concentration at 0.13–0.98 μmol/m^3^(Fig. 7). Although there was no significant correlation between *ISIP* expression and biomass at very low iron concentrations (0–0.05 μmol/m^3^, Fig. 7), the underlying reason may be the significant upregulation of *ISIPs* induced by extreme iron starvation (Additional file 1: Fig. S7), which masked the induction effect of chlorophyll fluorescence. Overall, this discovery further implies that, in the open ocean, besides environmental iron concentration, the surrounding chlorophyll fluorescence can also be sensed by diatom cells, thereby upregulating *ISIPs* to accelerate iron absorption.

Notably, the chlorophyll fluorescence intensity of two adjacent cells is 0.01 μmol/m^2^/s (lower than that in natural oceans due to the shading effect under experimental conditions). This also implies the potential involvement of chlorophyll fluorescence in the sexual reproduction, which has been reported to be triggered by red light in diatom [37] and required cell paring [38].

## Conclusion

This study demonstrated a cell density-dependent iron uptake regulation mechanism, which is mediated by chlorophyll fluorescence derived from neighboring cells. This result incites a reevaluation of the ecological and functional significance of red light, especially chlorophyll fluorescence, underwater. The detection of cell density-mediated iron uptake responses provides novel insights into the biotic signal sensing abilities of an ecological group of phytoplankton. However, further studies are required to investigate the possible relationships between red/far-red receptor protein phytochrome and iron uptake mechanism to gain a better and more robust understanding about the underlying molecular machinery.

## Methods

### Cell culture and treatment conditions

*P. tricornutum* was screened, identified [39], and grown in sterile artificial seawater (ASW) containing f/2 medium [40] at 20^o^C ± 1^o^C. The cultures were illuminated by fluorescent white lamps at an intensity of 80 μmol/m^2^/s under a light/dark cycle of 12/12 h.

For different cell density treatments, the algal cells were cultured under white light-emitting diode light (80 μmol/m^2^/s) till the mid-exponential growth phase and harvested by centrifugation at 2000×*g* for 5 min. Then, the cells were inoculated into fresh medium and the cell densities were adjusted to low (1.6×10^6^ cells/mL), medium (3.2×10^6^ cells/mL), and high (6.4×10^6^ cells/mL), cell density and cultured under light (80 μmol/m^2^/s), or dark conditions for 24 h. Subsequently, the cells were harvested by centrifugation at 6000×*g* for 1 min and quickly frozen with liquid nitrogen.

For different light treatments, the algal cells at various cell densities were cultured under blue light (BL, 460 nm, 80 μmol/m^2^/s) and white light (WL, 80 μmol/m^2^/s) for 24 h. Then, the cells were harvested by centrifugation at 6000×*g* for 1 min and quickly frozen with liquid nitrogen. Multi-cultivator MC 1000 (Photon Systems Instruments, Czech Republic) was used for eight different light quality treatments (at wavelengths of WL, 405 nm, 450 nm, 470 nm, 540 nm, 590 nm, 660 nm, and 730 nm, respectively, with the illumination of 80 μmol/m^2^/s).

For cell culture medium extraction treatment, the cells were harvested in a mid-exponential growth phase, and their density was adjusted to low (1.6×10^6^ cells/mL), medium (3.2×10^6^ cells/mL), and high (6.4×10^6^ cells/mL) and cultured under light (80 μmol/m^2^/s) for 24 h. After that, the algae cells were collected by centrifugation, and the upper medium was transferred to a clean sterile Erlenmeyer flask for later use. Then, the cell pellet was harvested and ultrasonicated on ice for 3 min, and the broken cells with low, medium, and high-cell densities were centrifuged and then collected. Clear, remix with the low, medium, and high upper layer medium obtained in the previous step. Use the resulting mixed solution to culture new algae cells in the dark for 24 h.

For “Sandwich” device treatment, bottle A and bottle C were black-coated on the outside and a red filter was plated between bottle B and A&C. *P. tricornutum* cells in bottle A and bottle C were excited by applying direct light in the *x*-axis to emit chlorophyll fluorescence in the *y*-axis. By altering the cell density in bottles A and C, the intensity of the excited fluorescence could be changed.

To obtain iron-deplete medium, PVC bottles were cleaned with HNO_3_ overnight. Then, ASW was prepared by using ddH_2_O, microwave-sterilized, and passed through a column containing Chelex 100 beads (Bio-Rad Laboratories) to remove the iron contaminant. To obtain an iron-replete medium, 11.7 μM FeCl_3_ was added to ASW. Cell growth was monitored at OD_730_ nm using a UV-VIS spectrophotometer (UV-1800; Shimadzu, Kyoto, Japan).

### Plasmid construction, nuclear transformation, and selection

The total RNA from *P. tricornutum* cells was isolated using RNA prep Pure Plant kit (polysaccharide and polyphenolic-rich) (Tiangen, Beijing, China) according to the manufacturer’s instructions. Subsequently, cDNA synthesis was performed using reverse transcription system (Takara, Beijing, China) according to the manufacturer’s instructions. The vectors for *ISIP2a* knockdown (Gene ID: 7200478) were generated by cloning the *ISIP2a* gene using primers (S-ISIP2a-F, S-ISIP2a-R; Additional file 1 Table S1) containing *Eco*RI and *Hind*III sites. The fragments were inserted into the multiple cloning sites of the pPha-T1 vector by *Eco*RI and *Hind*III restriction enzyme digestion for 15 min. The transgenic algal strain was constructed by biolistic transformation, and subsequent screening was performed as described earlier [41, 42]. The algal cells were transferred to a plate containing 100 μg/mL zeocin after they were allowed to recover for 24 h under low light. After 4 weeks, individual algal colonies were picked up and lysed for PCR analysis using T1yz-F and T1yz-R primers (Additional file 1: Table S1) to verify target gene integration. The positive colonies were cultured in a liquid medium containing 80 μg/mL zeocin and further verified using quantitative real-time PCR (qRT-PCR). To measure the growth curve of *ISIP2a*-Si strains, the cells were cultivated in iron-replete and iron-deplete ASW media, respectively. The iron-deplete ASW cultures were obtained using the trace metal clean method according to Kazamia et al. [12].

### qRT-PCR

The expression levels of *ISIP2a* and *ISIP1* genes were determined by qRT-PCR. The cDNA was obtained as described earlier and quantified by qRT-PCR using FastStart Essential DNA Green Master kit (Roche Diagnostics GmbH, Mannheim, Germany) and IQ5 multicolor real-time PCR detection system (Bio-Rad, Hercules, CA, USA) with Bio-Rad optical system software. The internal control was the 30S ribosomal protein subunit gene, and the primers used for qRT-PCR are shown in Additional file 1: Table S1.

### Chlorophyll fluorescence parameters

The photosynthetic activities of the algal cells were measured during the exponential growth phase using the Dual-PAM-100 fluorometer (Walz, Effeltrich, Germany). The algal cells were dark-adapted for 10 min, and the intrinsic fluorescence (F_0_) was measured. Subsequently, maximum fluorescence yield (F_m_) was detected when a saturation pulse was applied to calculate the F_v_/F_m_. Then, the light curve of Fluo and P700 was measured under stepwise illumination with increasing light intensities. The Fluo and P700 parameters were ascertained by applying a saturation pulse at the end of each light step, and the maximum fluorescence yield under illumination (*F*_m′_) was observed. The effective PSII quantum yield [Y(II)] was calculated using the formula: *Y*(II) = (*F*_m_–*F*)/*F*_m_, where *F* is the real-time fluorescence averaged for 0.2 s. Non-photochemical quenching (NPQ) was calculated as (*F*_m_–*F*_m′_)/*F*_m′_. The rETR(II) is a relative measure of the rate of electron transport (rate of charge separation at the PSII reaction centers), similar to rETR(I). For chlorophyll fluorescence measurement, the algal cells were illuminated with WL or BL, and plant lighting analyzer (PLA-20, EVERFINE Corporation, China) was used to measure fluorescence in the vertical direction.

### Pigment extraction and analysis

The *P. tricornutum* cultures were centrifuged at 6000×*g* for 1 min, and the algal cells obtained as pellet were desiccated using a vacuum freeze dryer. Then, the cells were ground (JX-FSTPRP-24 grinder, Shanghai, China), and the pigments were extracted using a solvent containing acetone/methanol (1:1). After 12 h of extraction at −20°C, the extracts were centrifuged at 10,000×*g* for 10 min at 4°C, and the supernatants were collected and filtered through a 0.22-μm nylon filter. Then, the supernatants were analyzed by high-performance liquid chromatography (HPLC) using a ZORBAX reverse-phase C_18_ column (Agilent1200, USA) at a constant temperature of 50°C and total flow rate of 0.8 mL/min. The assay was set as a linear gradient from mobile phase I (H_2_O: MeOH: ACN, 15:30:50) to phase II (MeOH: ACN, 15:85) during the first 15 min, phase II to phase III (H_2_O: MeOH: ACN: EtOAc,15:15:35:35) between 15 and 17 min, and phase III to phase IV (MeOH: EtOAc, 30:70) over the final 13 min. The compounds were examined based on absorbance at 443 nm. The pigment standards for chlorophyll a (Chl a), diadinoxanthin (Ddx), and diatoxanthin (Dtx) were obtained from Sigma-Aldrich (St. Louis, MO, USA), and de-epoxidation state (DEPS) was calculated as follows: DEPS = Dtx/(Ddx+Dtx).

### Intracellular iron quotas

To determine the intracellular iron concentrations, the *P. tricornutum* cells were washed thrice with a 10-mM EDTA, centrifuged, and freeze-dried using a vacuum freeze dryer. Subsequently, the cells were subjected to acid digestion to destroy the organic matter content. In brief, the cells were mixed with 5 mL of concentrated HNO_3_ and digested for 2 h at 150°C until the solution turned clear. Then, the solution was made up to 10 mL with ultrapure water. The iron content was determined in 2% HNO_3_ over a concentration range of 0.25–0.75 mg/L using calibration curves produced by an inductively coupled plasma optical emission spectrometer (ICP-OES, Optima 8000, PerkinElmer, USA).

### Mathematical model for chlorophyll fluorescence calculation

In order to calculate the chlorophyll fluorescence photon flux (CFPF) received at the single site of each cell, we build a mathematical model based on the radius of plastid *r* and attenuation coefficient of light underwater *K*_*d*_. Under laboratory conditions with incident blue light at 80 μmol/m^2^/s, we detected that the chlorophyll fluorescence photon flux density (CFPFD) in 1 mL cells was 2.418 × 10^−2^ μmol potons · m^−2^ · s^−1^ under the cell density *N* = 1.6 × 10^6^ cells · mL^−1^. So, we can convert the CFPFD to the CFPF emitted by per cell *u* = 9.07 × 10^−12^μmol potons · s^−1^. And then, we can calculate the CFPFD received per cell using the formula:

$$ \mathrm{CFPFD}=\mu \varOmega /\left(\frac{S_{plastid}}{2}\right)=u\frac{\pi {r}^2}{4\pi {R}^2}/\left(\frac{S_{plastid}}{2}\right) $$

Furthermore, considering the huge biomass and large-scale during diatom blooms, we build another mathematical model to calculate the CFPFD under stages with different cell densities (S1, S2 and S3) during blooms in actual oceans (Fig. b). In case of evenly distributed cells in real oceans, the groups of cells at the same distant *R* contribute equally to the cell at the center (Fig. a). If we further treat these cells as point light source, the CFPF emitted per unit cell *u* decays with spatial angle Ω and distance, and then, in an infinite space, the integral can be written as follows:

$$ \mathrm{CFPF}={\int}_0^{\infty}\left(4\pi {R}^2\cdotp dR\right)\cdotp N\cdotp u\cdotp \frac{\pi {r}^2}{4\pi {R}^2}\cdotp {e}^{-{K}_d\cdotp R}=\frac{Nu\pi {r}^2}{K_d} $$

where the radius of plastid *r* = 3 μm and attenuation coefficient of light underwater *K*_*d*_ = 0.57/*m* [43].

### In situ data analysis derived from Tara oceans

Correlations between normalized metatranscriptome (MetaT) expression of *ISIPs* and chlorophyll content among various Tara stations were conducted by GraphPad Prism 8.0.2. The *ISIPs* in each Tara station were expressed as a percentage of the total value of *ISIP1*, *ISIP2a*, *ISIP2b*, and *ISIP3* and normalized by the total diatom unigene expression, respectively, which were derived from the research by Caputi et al. [9], and also accessible at ENA under the accession number PRJEB6609. The observed dissolved iron concentrations and chlorophyll content in each Tara station are available at PANGAEA [44].

### Cis-acting element prediction

To predict the cis-acting elements in *P. tricornutum*, the 2000-bp upstream region of iron uptake-related genes in the *P. tricornutum* genome was extracted. Then, the upstream region sequences were submitted to the PlantCare (http://bioinformatics.psb.ugent.be/webtools/plantcare/html/) to predict cis-acting elements. The cis-acting elements related to light response were visualized using TBtools [45].

### Statistical analysis

All the results obtained in this study are expressed as mean values (*n* = 3). The data were first analyzed by one-way ANOVA and then subjected to post hoc analysis using Tukey’s test at an *α* = 0.05 significance level. All analyses were performed using SPSS 18.0 (SPSS Inc., Chicago, Il, USA). Details of statistical tests performed are in Additional File 2.

## Supplementary Information

**Additional file 1: Figs S1-S7, Table S1.** **Fig. S1.** Identification of ISIP2a knock down strain. (a) Growth analysis of WT, ISIP2a-S1 and ISIP2a-S2 under Fe deplete (-) and Fe replete (+) conditions. (b) relative expression of ISIP2a in WT, ISIP2a-S1 and ISIP2a-S2. Data was shown as mean values ± SD for three independent experiments. **Fig. S2.** Schematic illustration of the mathematical model for calculating the chlorophyll fluorescence photon flux density (CFPFD) received per cell. (a) Two adjacent cells. (b) Two cells with cellular distance at 10 μm. (c) Two cells with cellular distance at 100 μm. **Fig. S3.** Schematic illustration of the mathematical model for calculating the chlorophyll fluorescence photon flux (CFPF) during diatom blooms in oceans. (a) Evenly distributed cells in real water. The cell in the center can receive chlorophyll fluorescence from the whole space. (b) Percentage of CFPF versus upper limit of the integral. **Fig. S4.** Relative expression of flavodoxin under different cell density. The cells were adjusted to different cell density (L for low cell density, M for middle cell density and H for high cell density) and cultured at white light (WL), blue light (BL) and dark conditions. Data was shown as mean values ± SD for three independent experiments. **Fig. S5.** Relative expression of ISIP2a and ISIP1 in the experiments carried out with the following conditions: The cells at mid-exponential growth phase were harvested, resuspended with different sonicated medium of low cell density (L-CE), middle cell density (M-CE), and high cell density (H-CE) and cultured at dark for 24 h. Data was shown as mean values ± SD for three independent experiments. **Fig. S6.** Prediction of cis-acting element in the iron uptake related genes in P. tricornutum. **Fig. S7.** Correlations between MetaT expression of ISIPs and iron concentration among various Tara stations. The ISIPs in each Tara stations were expressed as a percentage of the total value of ISIP1, ISIP2a, ISIP2b and ISIP3, and normalized by the total diatom unigene expression, respectively. Pearson correlation coefficients (Pearson r) and their statistical significance (p value) are indicated in graph. **Table S1.** Primer list

**Additional file 2.** Statistical tests

**Additional file 3.** Raw data

## Data Availability

The datasets used and/or analyzed and the materials in this study are available from the corresponding author on reasonable request. Metatranscriptome data mentioned in Fig. 7 are archived at ENA under the accession number PRJEB6609 [46]. Raw data are listed in Additional File 3.

## Abbreviations

HNLC: High-nutrient low-chlorophyll
MetaT: Metatranscriptome
CFPF: Chlorophyll fluorescence photon flux
CFPFD: Chlorophyll fluorescence photon flux density
