# Supplementary material for: Chlorophyll fluorescence as a light signal enhances iron uptake by the marine diatom Phaeodactylum tricornutum under high-cell density conditions
Source: BMC Biol. 2021 Nov 23;19:249. doi: 10.1186/s12915-021-01177-z (PMC8609858; doi:10.1186/s12915-021-01177-z)
Supplement: Supplementary file 1 — Additional file 1: Figs S1-S7, Table S1. Fig. S1. Identification of ISIP2a knock down strain. (a) Growth analysis of WT, ISIP2a-S1 and ISIP2a-S2 under Fe deplete (-) and Fe replete (+) conditions. (b) relative expression of ISIP2a in WT, ISIP2a-S1 and ISIP2a-S2. Data was shown as mean values ± SD for three independent experiments. Fig. S2. Schematic illustration of the mathematical model for calculating the chlorophyll fluorescence photon flux density (CFPFD) received per cell. (a) Two adjacent cells. (b) Two cells with cellular distance at 10 μm. (c) Two cells with cellular distance at 100 μm. Fig. S3. Schematic illustration of the mathematical model for calculating the chlorophyll fluorescence photon flux (CFPF) during diatom blooms in oceans. (a) Evenly distributed cells in real water. The cell in the center can receive chlorophyll fluorescence from the whole space. (b) Percentage of CFPF versus upper limit of the integral. Fig. S4. Relative expression of flavodoxin under different cell density. The cells were adjusted to different cell density (L for low cell density, M for middle cell density and H for high cell density) and cultured at white light (WL), blue light (BL) and dark conditions. Data was shown as mean values ± SD for three independent experiments. Fig. S5. Relative expression of ISIP2a and ISIP1 in the experiments carried out with the following conditions: The cells at mid-exponential growth phase were harvested, resuspended with different sonicated medium of low cell density (L-CE), middle cell density (M-CE), and high cell density (H-CE) and cultured at dark for 24 h. Data was shown as mean values ± SD for three independent experiments. Fig. S6. Prediction of cis-acting element in the iron uptake related genes in P. tricornutum. Fig. S7. Correlations between MetaT expression of ISIPs and iron concentration among various Tara stations. The ISIPs in each Tara stations were expressed as a percentage of the total value of ISIP1, ISIP2a, ISIP2b and ISI [file 12915_2021_1177_MOESM1_ESM.docx]

**Additional file 1**

**Fig. S1 Identification of *ISIP2a* knock down strain.** (a) Growth analysis of WT, ISIP2a-S1 and ISIP2a-S2 under Fe deplete (-) and Fe replete (+) conditions. (b) relative expression of *ISIP2a* in WT, ISIP2a-S1 and ISIP2a-S2. Data was shown as mean values ± SD for three independent experiments.


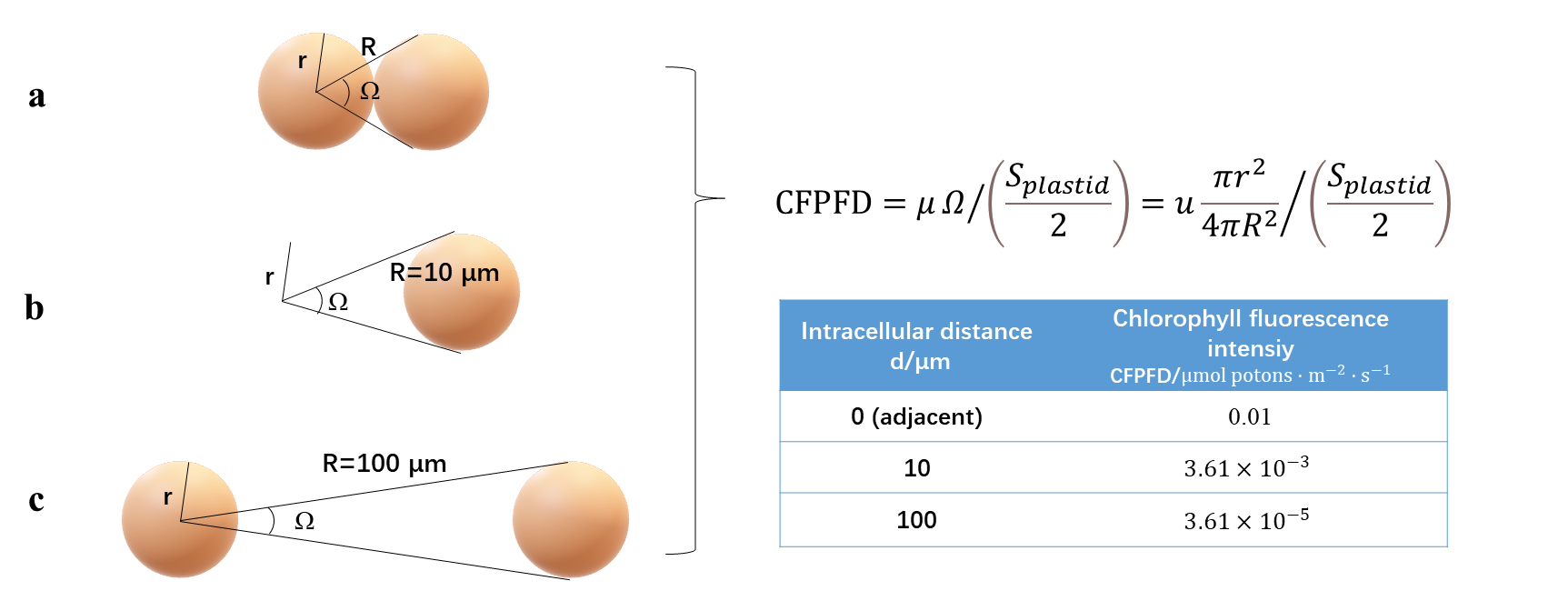


**Fig. S2 Schematic illustration of the mathematical model for estimating the chlorophyll fluorescence photon flux density (CFPFD) received per cell.** (a) Two adjacent cells. (b) Two cells with cellular distance at 10 μm. (c) Two cells with cellular distance at 100 μm.


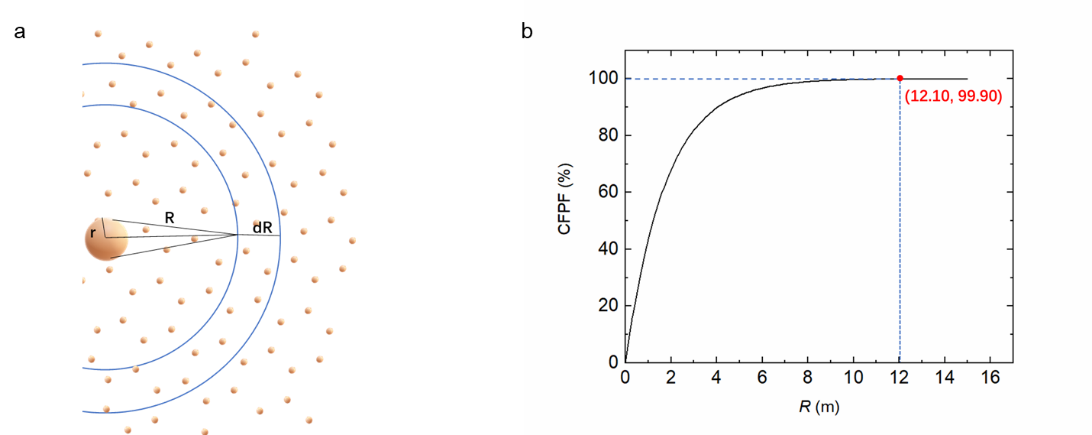


**Fig. S3 Schematic illustration of the mathematical model for estimating the chlorophyll fluorescence photon flux (CFPF) during diatom blooms in oceans.** (a) Evenly distributed cells in real water. The cell in the center can receive chlorophyll fluorescence from the whole space. (b) Percentage of CFPF versus upper limit of the integral.

**Fig. S4 Relative expression of *flavodoxin* under different cell density.** The cells were adjusted to different cell density (L for low cell density, M for middle cell density and H for high cell density) and cultured at white light (WL), blue light (BL) and dark conditions. Data was shown as mean values ± SD for three independent experiments.

**Fig. S5 Relative expression of *ISIP2a* and *ISIP1* in the experiments carried out with the following conditions:** The cells at mid-exponential growth phase were harvested, resuspended with different sonicated medium of low cell density (L-CE), middle cell density (M-CE), and high cell density (H-CE) and cultured at dark for 24 h. Data was shown as mean values ± SD for three independent experiments.


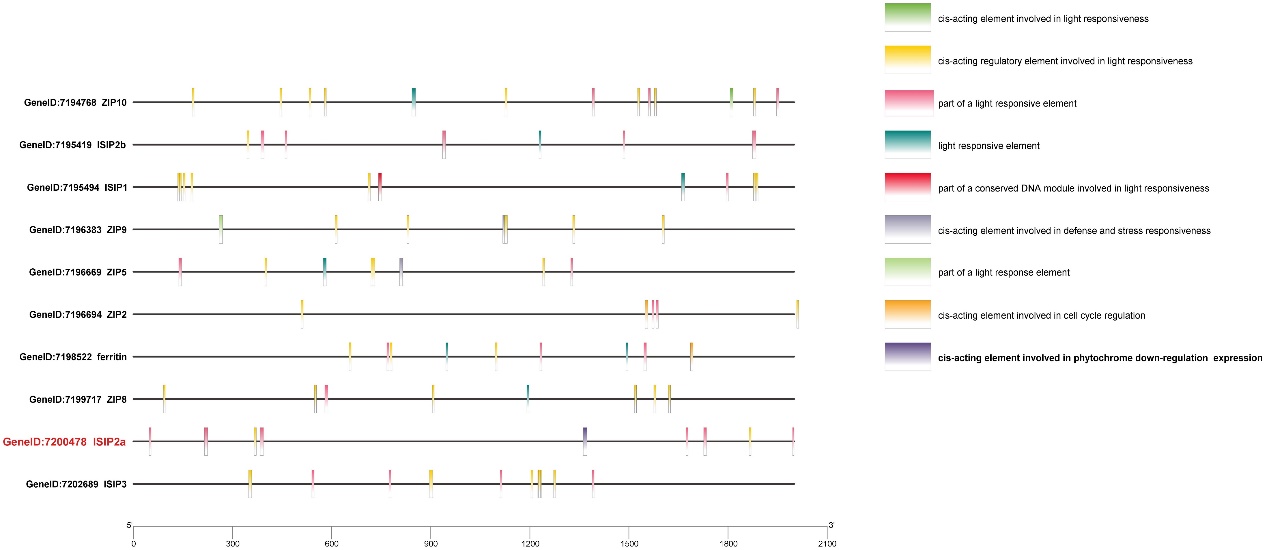


**Fig. S6 Prediction of cis-acting element in the iron uptake related genes in *P. tricornutum.***


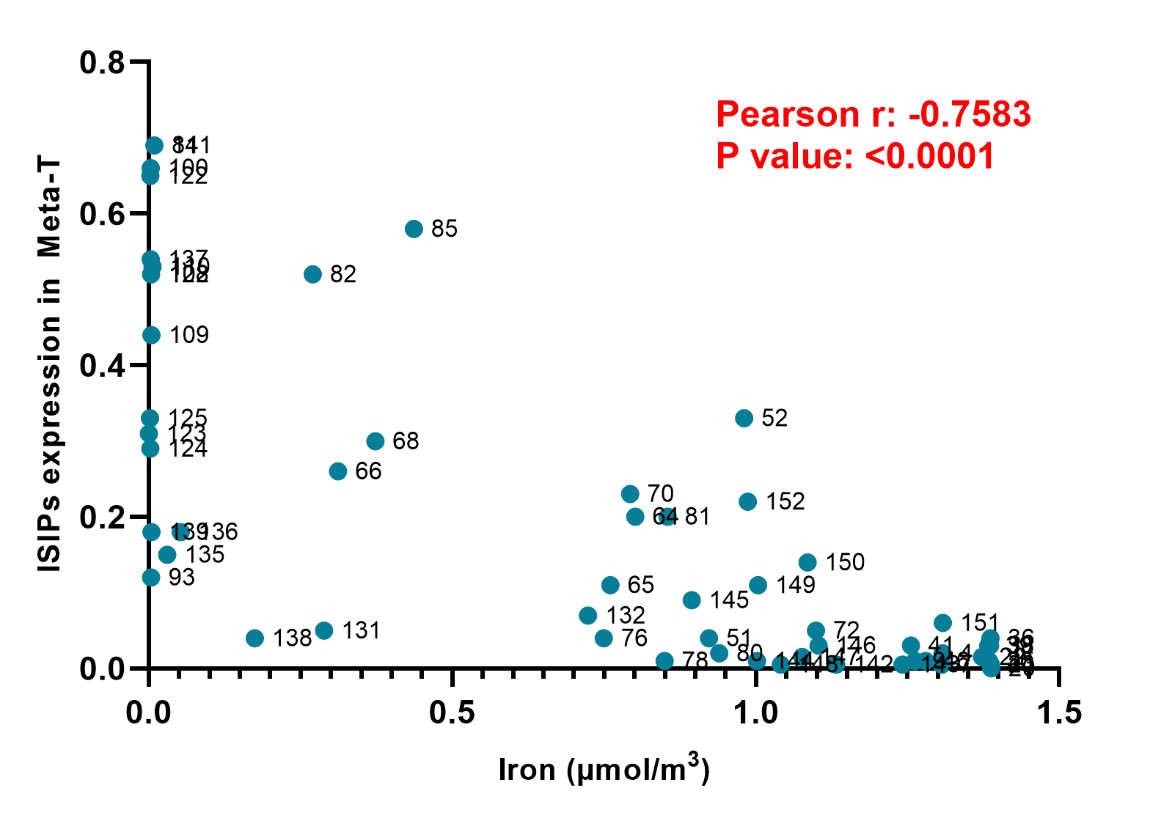


**Fig.S7 Correlations between MetaT expression of *ISIPs* and iron concentration among various Tara stations.** The *ISIPs* in each Tara stations were expressed as a percentage of the total value of *ISIP1, ISIP2a, ISIP2b and ISIP3*, and normalized by the total diatom unigene expression, respectively. Pearson correlation coefficients (Pearson r) and their statistical significance (p value) are indicated in graph.

Table S1 Primer list

| **Primers for qRT-PCR** | |
| --- | --- |
| RT-Fla-F | CGTGCTCTACTACAACCA |
| RT-Fla-R | CCGTCCCTAATACTCTTC |
| RT-ISIP2a-F | GTGCCACGAAAGAGAAAA |
| RT-ISIP2a-R | CAAAAGCATCGAAAACGG |
| Rt-ISIP1-F | AACTCCTCTTGTCGGCTT |
| Rt-ISIP1-R | GGTGCTTCCTCGATATCC |
| RPS-F | CGAAGTCAACCAGGAAACCAA |
| RPS-R | GTGCAAGAGACCGGACATACC |
| **Primers for transgenetic algae validation** | |
| T1yz-F | TGAATTGACTCACGGTCTT |
| T1yz-R | AGGATTTGCTGTTTACGG |
| **Primers for FTN-Si plasmid construction** | |
| S-ISIP2a-F | GCTCTAGA CCAAGTCATTCGCTGTG |
| S-ISIP2a-R | CCGGAATTC CAGGATTACCACCCACC |
